# The Memory Aid study: protocol for a randomized controlled clinical trial evaluating the effect of computer-based working memory training in elderly patients with mild cognitive impairment (MCI)

**DOI:** 10.1186/1745-6215-15-156

**Published:** 2014-05-03

**Authors:** Marianne M Flak, Susanne S Hernes, Jon Skranes, Gro CC Løhaugen

**Affiliations:** 1 Department of Medicine, Geriatric Unity, The Memory Clinic, Sørlandet Hospital, Arendal, Norway; 2 Department of Pediatrics, Sørlandet Hospital, Arendal, Norway; 3 Department of Laboratory Medicine, Children’s and Women’s Health, Norwegian University of Science and Technology, Trondheim, Norway

## Abstract

**Background:**

Mild cognitive impairment (MCI) is a condition characterized by memory problems that are more severe than the normal cognitive changes due to aging, but less severe than dementia. Reduced working memory (WM) is regarded as one of the core symptoms of an MCI condition. Recent studies have indicated that WM can be improved through computer-based training. The objective of this study is to evaluate if WM training is effective in improving cognitive function in elderly patients with MCI, and if cognitive training induces structural changes in the white and gray matter of the brain, as assessed by structural MRI.

**Methods/Designs:**

The proposed study is a blinded, randomized, controlled trail that will include 90 elderly patients diagnosed with MCI at a hospital-based memory clinic. The participants will be randomized to either a training program or a placebo version of the program. The intervention is computerized WM training performed for 45 minutes of 25 sessions over 5 weeks. The placebo version is identical in duration but is non-adaptive in the difficulty level of the tasks. Neuropsychological assessment and structural MRI will be performed before and 1 month after training, and at a 5-month folllow-up.

**Discussion:**

If computer-based training results in positive changes to memory functions in patients with MCI this may represent a new, cost-effective treatment for MCI. Secondly, evaluation of any training-induced structural changes to gray or white matter will improve the current understanding of the mechanisms behind effective cognitive interventions in patients with MCI.

**Trial registration:**

ClinicalTrials.gov NCT01991405. November 18, 2013.

## Background

Mild cognitive impairment (MCI) is a clinical condition characterized of a reduction in memory and/or other cognitive processes that are insufficiently severe to be diagnosed as dementia, but are more pronounced than the cognitive decline associated with normal aging. In Norway, MCI and dementia represent major health concerns, which will be even more pronounced in the future because of the demographic shift that is resulting in an increased number of elderly citizens. The economic burden of these disorders both for the individual and for society is a heavy one [1,2]. Currently, no treatment is available for enhancing memory function or preventing further decline in patients with MCI. Impairment of memory and learning, including the working memory (WM) capacity, characterizes an MCI condition [3].

WM is a theoretical construct referring to the structures and processes associated with our ability to keep information in mind, while we actively use and respond to the information [4,5]. Individuals with WM deficits have trouble remembering information even for a few seconds, find it difficult to keep track of what they are doing as they work, or forget what they are supposed to retrieve when sent on an errand [6]. Previously, a person’s WM capacity was presumed to be constant and unmodifiable by stimulation or other external factors. Recently, the 'neuroplasticity paradigm' has gained support, and a growing body of research has focused on the brain’s modification capabilities. The notion that mentally stimulating activities may affect cognitive processes, including memory, has also been linked to a 'use it or lose it' hypothesis [7].

Different interventions have been developed to try to enhance or preserve cognitive function [8]. Strategic interventions (by which an individual learns strategies to circumvent and compensate for the cognitive deficits) may be difficult for patients with MCI patients because of their memory problems. We use the definition suggested by Jolles and Crone [9] of cognitive training as the process of improving cognitive functioning by means of practice and/or intentional instruction. The intervention method in this study can be classified as a process-based approach, which involves repeated performance (that is, practice) of demanding (WM) tasks, for which an underlying change in neural correlates is hypothesized [9,10]. WM capacity is associated with the organization of the white matter tracts that constitute the network between the parietal and the frontal lobes [11]. This assumption has been supported by the finding that adaptive WM training causes structural changes of the nerve tracts in the brain [12-14]. It is plausible to expect that performance gain in WM would improve various everyday activities that place high demands on WM capacity [9,15-17].

Positive effects of WM training have been reported in diverse clinical groups, including children with attention deficit hyperactivity disorder (AD/HD), as well as preschool children and youths born preterm with very low birth weight [18-32]. Reviews have reported moderate to large beneficial effects of cognitive exercises on memory-related outcomes [8,10,33]. Enhancing the function in specific domains by training the impaired skill(s) demonstrate the greatest overall effect on functioning compared with non-training methods such as memory strategies and compensatory strategies in patients with Alzheimer’s disease [8]. Several studies have reported improved performance on tasks included in the intervention (trained tasks) as well as improvement on non-trained WM tasks and other cognitive tasks that require spatial skills and involve complex problem-solving skills. However, there is a lack of studies on effective training interventions for MCI patients, and the few randomized controlled trials (RCTs) that exist have shown divergent results [16,33,34]. In addition, the existing studies have the limitations of non-standardized intervention methods and low statistical power, rendering conclusions on these matters challenging. It is therefore of great importance to perform RCTs investigating the effects of specific cognitive interventions in elderly patients with MCI who are at high risk of developing dementia. In addition, the proposed study seeks to investigate individual differences in training outcome and its relation to dementia biomarkers such as tau, phospho-tau, and β-amyloid proteins as well as candidate genes such as *APOE* and *LMX1A*. A recent study by Bellander *et al*. [35] indicates that certain combinations of the different alleles of the *LMX1A* gene might predict those healthy adults most likely to benefit from WM training. This gene is involved in both development and maintenance of dopamine-producing neurons, which are related to WM function [36-40]. If this applies to the MCI group, such a biomarker could be a marker for the effects of the intervention and thereby correlate with the feasibility of this particular intervention. This article describes a study that it aimed to increase knowledge of the neuropsychological status in patients with MCI, as well as the feasibility and possible effects of a cognitive training intervention.

## Methods/Design

### Objectives

The primary objective of this blinded RCT is to evaluate the hypothesis that standardized computer-based WM training is effective in improving WM capacity over time in patients diagnosed with MCI. Second, we want to assess whether computer-based adaptive WM training in patients with MCI has transfer effects to other cognitive functions, including memory, attention, and executive functions, as well as activities of daily living, measured by a battery of neuropsychological tests. Third, any effects of WM training on the structural changes in white and gray matter of the brain will be investigated, measured by multimodal magnetic resonance imaging. Finally, possible genetic biomarkers (*LMX1A* and *APOE* genotypes) related to a positive effect or lack of effect of the training will be examined.

Study approval was given by the Norwegian regional committee for medical and health research ethics, south-eastern region (reference number 2013/410/REK Sør-øst). The study will be reported in accordance with both the CONSORT statement and the CONSORT statement for non-pharmacological interventions [41,42].

### Participants

In total, 90 patients diagnosed with MCI recruited from memory clinics at Sørlandet Hospital, Arendal, The Hospital of Telemark, and Oslo University Hospital will be included in the study. The patients will undergo standard medical examination and neuropsychological testing to ensure correct diagnosis of MCI. Informed consent will be obtained from each participant.

### Inclusion and exclusion criteria

Patients who meet the Peterson diagnostic criteria of MCI [2]: 1) memory problems, preferably confirmed by an informant, 2) memory impairment in accordance with age and education, 3) preserved general cognitive function, 4) intact activities of daily living, and (5) absence of dementia (in accordance with the ICD-10/DMS-IV criteria). Patients who have had head trauma with post-traumatic loss of consciousness for at least 30 minutes during their lifespan, or who have loss of senses (blindness, deafness) or photosensitive epilepsy or who are unsuitable for magnetic resonance imaging because of implanted metal foreign bodies or severe claustrophobia will be excluded from the study.

### Outcome measures and assessment procedures

Baseline assessment will be completed prior to randomization and the first intervention session.

### Neuropsychological assessment

The neuropsychological/cognitive evaluation includes the administration of several commonly used measures (tests, interview, and questionnaires) of cognitive abilities and daily functions. Measures of the patient's cognitive status (intelligence, memory, processing speed, attention, and executive functions) will include a wide range of standardized neuropsychological tests, as well as tests assessing prefrontal functions, as these may mediate WM decline [43]. Measures are administered as a combination of paper-and-pencil tests and a computer-administered task. The sensitivity and specificity of the assessment methods have been described previously [44,45]. To correct for learning effects on repeated tests, alternative test versions or forms will be used when possible. The selected neuropsychological tests and assessed functions is described in Table 1.

**Table 1:** Summary of assessed cognitive functions and tests

**Function:**	**Tests:**
General cognitive ability (IQ)	WAIS-IV
Auditory and visual WM	Digit Span, Spatial Span/Span Board and Letter-Number Sequencing, PASAT
Verbal and visual learning, immediate and delayed memory	WMS-III, CVLT-II
Attention and executive functions	D-KEFS subtests including: Trails 1 to 5, Color Word Interference, Tower, Verbal fluency, Design fluency. Supplemental test: Iowa Gambling Task

### Interview and questionnaires

The following interviews and questionnaires will be used.

• Vineland Adaptive Behavior Scales, second edition (interview). This structured interview measures adaptive abilities in three broad domains: communication, activities of daily living, and social skills.

• Behavior Rating Inventory of Executive Functions Adult version (BRIEF-A). This questionnaire measures nine domains of executive functions (inhibition, shifting, emotional control, self monitoring, initiating, WM, planning/organizing, task monitoring, and organization of materials) through a self-reported and an informant version.

• Behavioral assessment of the Dysexecutive Syndrome Questionnaire (DEX). Assesses problems in executive function through a self-report and an informant version.

### Magnetic resonance brain imaging

The patients will undergo brain MRI scanning at baseline and at 1 and 4 months post-training, using an optimized protocol. Following a pilot scan, a three-dimensional (3D) magnetization-prepared rapid gradient echo (MP-RAGE) scan will be performed (sagittal, echo time 3.47, repetition time 2400, TI 1000, flip angle (FA) 8 degrees, 1.2 mm resolution covering the whole brain).

Diffusion tensor imaging (DTI) scan will be performed (axial (non-oblique), 68 slices, field of view = 240 mm, 2.5 mm slices without gap; 2.5 × 2.5 mm in-plane resolution; repetition time9.500, echo time 91, two averages, diffusion (b = 0.1000), 30 diffusion directions. In addition a 3D-T2 space dark fluid (FLAIR) image (sagittal, echo time 335, repetition time 5000, TI 1800, turbo factor 242, 1.2 mm resolution covering the whole brain) and a T2* image (axial TE 25, TR 830, flip angle 20 degrees) will also be acquired during the initial scan for clinical evaluation to exclude microbleeds. Total scanning time for the initial scan will be close to 30 minutes. The follow-up scans will be under 25 minutes. All scans will be reviewed qualitatively by a radiologist to screen for possible brain lesions or structural abnormalities. By using tract-based spatial statistics (TBSS), the radiologist will measure fractional anisotropy, and mean diffusivity values in central white matter tracts before and after training. Automated morphometry to evaluate cortical thickness, surface area, and total gray and white matter volumes, as well as deep nuclei volumes will be performed.

### Biological/biochemical samples, study biobank, and DNA collection

One oral cotton swab will be collected for genotype analyses for *APOE* and *LMX1A* alleles. DNA will be extracted from saliva collected using an Oragene Self collection Kit (DAN Genoteck, Inc. Ottawa, Ontario, Canada). Genomic DNA will be subjected to Restriction Fragment Length Polymorphism analyses (RFLP-PCR). Approximately 3 ng of genomic DNA for *LMX1A* will be amplified by PCR using the primer LMX-5′: 5′-CTCGCCTCCAGGAA TGGGTGTTGTA-3′and LMX-3′: 5′-GCCACGAGGAACTTGTGAGAGGGTT-3′ with the following conditions: denaturation at 94°C for 5 minutes, followed by 25 cycles at 94°C for 30 seconds, annealing at 64°C for 30 seconds and extending at 72°C for 30 seconds. Then 15 μl of the amplification PCR products will be digested directly by 2.5 U of the restriction enzyme *Msl*I (R0571S, New England Biolabs, Beverly, MA, USA) for 2 hours at 37°C. The digested PCR products will then be analyzed on 4% agarose gel, and visualized using GelGreen™ Nucleic Acid Gel Stain (89139–144, Biotium, Hayward, CA, USA).

### Primary outcome measure

Primary outcome measure is the spatial span board task from the Wechsler Memory Scale, third edition (WMS-III).

### Secondary outcome measures

Secondary outcome measureszare the structural MRI changes, the results on neuropsychological test measuring verbal and visual learning and memory, and the response pattern on the questionnaires.

#### Intervention

The intervention method is classified as an adaptive, intensive, and standardized computerized WM training program (Cogmed®), developed by Professor T. Klingberg at the Karolinska Institute, Stockholm, Sweden [29,46]. Both the adaptive and the 'placebo' training groups will use the program approximately 30 to 40 minutes a day, 5 days a week for 5 weeks. The training is considered 'adaptive', which means that the difficulty level of the tasks increases during the sessions according to the individual level of mastering for each participant, making the patient work at their maximum capacity at all times [46]. This is the core element of the hypothesized effectiveness of the intervention. The placebo group will use a non-adaptive (fixed low-level training) version of the training program. The interface of both the placebo and the training version is identical, but the difficulty level in the placebo version does not increase adaptively as the patient performance improves. The study involves three examinations at the following time points: baseline before training, retesting about 1 month after training completion, and follow-up retesting four months after training completion in order to examine the longer-term effects, see Table 2 for details.

**Table 2:** Assessment measures at baseline and post-intervention evaluations

**Assessment tool**	**Baseline evaluation**	**Post-intervention evaluation**	
		**1: 1 month after training**	**2: 4 months after training**
Medical examination	X		
MR caput	X	X	X
Spinal fluid	X		
DNA sampling	X		
WAIS-IV	X		
WMS-III	X	X	X
Digit span	X	X	X
Spatial span	X	X	X
Letter-Number Sequencing	X	X	X
CVLT-II	X	X	X
D-KEFS	X	X	X
IGT	X		
Vineland	X		X
BRIEF-A	X	X	X
DEX	X	X	X

#### Randomization

Participants in the study are randomly allocated to either the adaptive training group or the non-adaptive (placebo) training group after the baseline assessment. The patients randomized to the placebo group will be offered the adaptive training after the 4-month follow-up examination. The neuropsychologist and radiologist carrying out the assessments are blinded to group adherence.

Both the adaptive and the placebo training groups undergo a 25-session standardized computerized WM training within the same time period (all participants start training/placebo at the same time), differing only in the adaptability of the training. During the training phase, weekly follow-ups by standardized phone interviews are scheduled for all participants regardless of group affiliation. After the initial training phase, the participants are examined at 1 and 4 months after finalizing their training. All participant contact is logged in order to discover potential performance bias. In addition to the research team, the patients are followed by their primary physician, who will tend to all non-trial-related issues.

### Sample size and power calculation

Using pre-training and post-training test scores obtained by Brehmer *et al*. [47], 72 patients are needed for inclusion in the study to obtain a statistical power of 80%. Allowing for a maximum dropout rate of 20%, the number of study subjects needed for inclusion has been set to 90 patients with MCI. Power calculations are based on the primary outcome measure, which is change in WM capacity assessed by neuropsychological tests and behavioral inventories that assess WM and related functions. The statistical power measures will be calculated using Sample Power 2.0.

### Statistical analysis

Performance gain in the assessment scores related to WM function from baseline is the primary outcome of interest. Participants' daily performance on the WM tasks within the program will be aggregated into one *t*-standardized performance score. Weekly performance scores will be used for analysis. A repeated-measure ANOVA will be conducted to investigate performance gain during the training period. One-way ANOVA will be conducted separately for the assessment criterion and transfer tasks and the rating scales of functioning, to examine potential baseline differences between the intervention and the placebo groups. To determine differences in training-related changes, a general linear mixed model with appropriate covariates will be conducted (baseline, post-training, and follow-up), and within-subject factors will be used for the cognitive tasks and assessment data. For all analyses, α levels will be set to 0.05 and effect sizes refer to partial η-square values. With regard to cerebral MRI, the DTI images will be analyzed using the software FSL with [48] to provide information on any changes in white matter microstructure with training. Automated morphometry to evaluate cortical thickness and surface area, total gray and white matter volumes, and deep nuclei volumes will be performed using the freely available FreeSurfer software program, v5.1 [49]. Complete-case analysis will be used where missing data are excluded from the analysis. A fully detailed statistical analysis will be performed before unmasking of the data.

#### Dissemination plan

The dissemination plan includes five scientific papers that will be submitted to peer-reviewed medical journals:

1. Computer-based adaptive working memory training on cognitive functioning in elderly patients with mild cognitive impairment assessed by neuropsychological tests. A randomized double-blind controlled study.

2. Does cognitive training in patients with Mild Cognitive Impairment relate to structural changes in the white and gray matter of the brain?

3. Do patients with MCI with a particular genotype for the *LMX1A* gene (i.e, AA alleles) or for *APOE* (i.e. non-ϵ4) have stronger training effects after adaptive working memory training?

4. Does computer -based working memory training have secondary effects on executive function and activities of daily living in elderly patients with MCI? A randomized, double-blind controlled study.

5. Long-term follow-up of elderly patients with mild cognitive impairment after completion of an adaptive, computerized, standardized, and intensive working memory program: neuropsychological and daily-life functioning.

The first publication will be submitted at the end of 2014, and the other articles will be submitted before the project ends in 2017. In addition, the results will be disseminated at international conferences and national forums, and in the popular press to target a wide range of groups, including researchers, healthcare providers, and health-care professionals, as well as the general public. The results will be disseminated regardless of the magnitude or direction of effect.

## Discussion

At present, there is no known cure for memory decline in the elderly or other cognitive deficits that follows MCI. Discovery of a method capable of enhancing memory, or postponing further decline in WM function in patients in the early stages of dementia, would be of great importance for a large and growing patient group.

The strengths of this trial are the extensive assessment of the participants on several levels, including gene samples, quantitative brain imaging, and neuropsychological testing. We hope the assessment will provide new and important information on the memory abilities and daily life functioning in elderly patients with MCI. This extensive assessment is relevant because of the complex nature of the etiology and behavioral outcomes of memory decline in elderly patients, and the heterogeneity of the cognitive phenotypes seen in memory clinics. The proposed study will be the first to investigate the possible mediating influence of biomarkers on the effects of cognitive training in patients with MCI. Such biomarkers may contribute in prediction of training effects. Furthermore, identification of a genetic marker to assess WM trainability would be an important finding that would allow cognitive intervention to be offered to those best able to benefit from it. This would also be of benefit to society.

Participating in this study involves no negative effects for the patients; but it may help the patients to improve their everyday functioning, and perhaps also to postpone further decline in WM function. The study also seeks to identify cognitive reserves in these patients, as such reserves seem to play an important role in the development of cognitive impairment [50]. Currently, there is a lack of knowledge of the effects of adaptive, standardized and intensive computerized WM training on individuals with MCI, and this study will be able to provide new and unique knowledge. If WM training can be shown to have positive effects on cognitive and daily-life functioning in people with MCI, a new approach to treatment of memory decline may be feasible.

## Trial status

The trial started in August 2013, and is currently enrolling participants.

## Abbreviations

BRIEF-A: Brief Rating Inventory of Executive Functions Adult version; CVLT-II: California Verbal Learning Test, second edition; D-KEFS: Delis-Kaplan Executive Function System; DEX: Behavioral assessment of the Dysexecutive Syndrome Questionnaire; IGT: Iowa Gambling Task; MCI: Mild Cognitive Impairment; Vineland: The Vineland Adaptive Behavior Scales, second edition (interview edition); WAIS-IV: Wechsler Adult Intelligence Scale, fourth edition; WMS-III: Wechsler Memory Scale, third edition.

## Competing interests

The authors declare that they have no competing interests.

## Authors’ contributions

MMF: conception and design, data collection and analysis, manuscript writing, and final approval of the manuscript. SSH: conception and design, data collection and analysis, manuscript writing, and final approval of the manuscript. JS: conception and design, data interpretation, manuscript writing, critical revision, and final approval of the manuscript. GCCL: conception and design, manuscript writing, critical revision, and final approval of the manuscript. All authors read and approved the final manuscript.
