# Erratum to: ‘The Memory Aid study: protocol for a randomized controlled clinical trial evaluating the effect of computer-based working memory training in elderly patients with mild cognitive impairment (MCI)’

**DOI:** 10.1186/s13063-016-1180-0

**Published:** 2016-01-20

**Authors:** Marianne M. Flak, Susanne S. Hernes, Linda Chang, Thomas Ernst, Vanessa Douet, Jon Skranes, Gro C. C. Løhaugen

**Affiliations:** Department of Medicine, Geriatric Unit, The Memory Clinic, Sørlandet Hospital, Arendal, Norway; Department of Medicine, John A. Burns School of Medicine, The Queen’s Medical Center, Honolulu, HI USA; Department of Pediatrics, Sørlandet Hospital, Arendal, Norway; Department of Laboratory Medicine, Children’s and Women’s Health, Norwegian University of Science and Technology, Trondheim, Norway

Unfortunately, the original version of this article [1] contained an error. Three authors were omitted from the author list and have been included here so that the complete author list reads as: Marianne M Flak^1,2^*, Susanne S. Hernes^1^, Linda Chang^2^, Thomas Ernst^2^, Vanessa Douet^2^, Jon Skranes^3,4^, and Gro C. C. Løhaugen ^3,4^.

Another consequence of the first mistake is that the sections about MRI (4^th^ section page 3) and genotyping (last section on page 3 and first section on page 4) contain some errors.

The MRI section should read as follows:

Magnetic resonance brain imaging

The patients will undergo brain MRI scanning at baseline and at 1 and 4 months post-training, using an optimized protocol. Following a pilot scan, a three-dimensional (3D) magnetization-prepared rapid gradient echo (MP-RAGE) scan will be performed (sagittal, echo time 3.47 ms, repetition time 2400 ms, TI 1000 ms, flip angle (FA) 8 degrees, 1.2 mm resolution covering the whole brain).

Diffusion tensor imaging (DTI) scan will be performed (axial (non-oblique), 68 slices, field of view = 240 mm, 2.5 mm slices without gap; 2.5 × 2.5 mm in-plane resolution; repetition time 9.500 ms, echo time 91 ms, two averages, diffusion {b = 0 and 1000 s/mm^2^}, 30 diffusion directions. In addition, a 3D-T2 space dark fluid (FLAIR) image (sagittal, echo time 335 ms, repetition time 5000 ms, TI 1800 ms, turbo factor 242, 1.2 mm resolution covering the whole brain) and a T2* image (axial TE 25 ms, TR 830 ms, flip angle 20 degrees) will be acquired during the initial scan for clinical evaluation to exclude microbleeds. Total scanning time for the initial scan will be close to 30 minutes. The follow-up scans will be under 25 minutes. All scans will be reviewed qualitatively by a radiologist to screen for possible brain lesions or structural abnormalities. By using tract-based spatial statistics (TBSS or DTIStudio) axial, medial and mean diffusivity values will be measured in white matter tracts involved in the working memory network before and after training. Automated morphometry to evaluate cortical thickness, surface area, and total gray and white matter volumes, as well as deep nuclei volumes will be performed using Freesurfer.

The section about genotyping should read as follows:

Biological/Biochemical Samples, Study Biobank and DNA Collection

DNA will be extracted from saliva collected in Oragene Self collection Kit (DAN Genoteck, Inc. Ottawa, Ontario, Canada). Genomic DNA will be subjected to Restriction Fragment Length Polymorphism analyses (RFLP-PCR) for genotype analyses of *APOE*ε (rs429358 and rs7412) and *LMX1A* (rs4657412). Approximately 3 ng of genomic DNA will be amplified by PCR using the primers LMX-5’:5’-CTCGCCTCCAGGAA TGGGTGTTGTA-3’ and LMX-3’: 5’-GCCACGAGGAACTTGTGAGAGGGTT-3’ for *LMX1A* and APO-5’ and APO-3’ for *APOE*ε (Andres et al., 2011) with the following conditions: denaturation at 94 °C for 5 minutes, followed by 30 cycles at 94 °C for 30 seconds, annealing at 64 °C for 30 seconds and extending at 72 °C for 30 seconds. Then 15 μl of the amplification PCR products will be digested directly by 2.5 U of restriction enzymes MslI (R0571S, New England Biolabs, Beverly, MA) for 2 hours at 37 °C, and by *Hae*II (R0107S, New England Biolabs, Beverly, MA, USA) and *Afl*III (R0541S, New England Biolabs, Beverly, MA, USA) overnight at 37 °C. The digested PCR products will then be analyzed on 4 % agarose gel and visualized using GelGreen™ Nucleic Acid Gel Stain (89139–144, Biotium, Hayward, CA).
